# Supplementary material for: Splice-Junction-Based Mapping of Alternative Isoforms in the Human Proteome
Source: Cell Rep. Author manuscript; Available in PMC 2020 Jan 15. (PMC6961840; doi:10.1016/j.celrep.2019.11.026)
Supplement: 2 [file NIHMS1546469-supplement-2.zip › DF1/PXD006675/Aorta/Aorta_20_DVL3_SLDDFHLSIHSDMAAIVK.pdf]

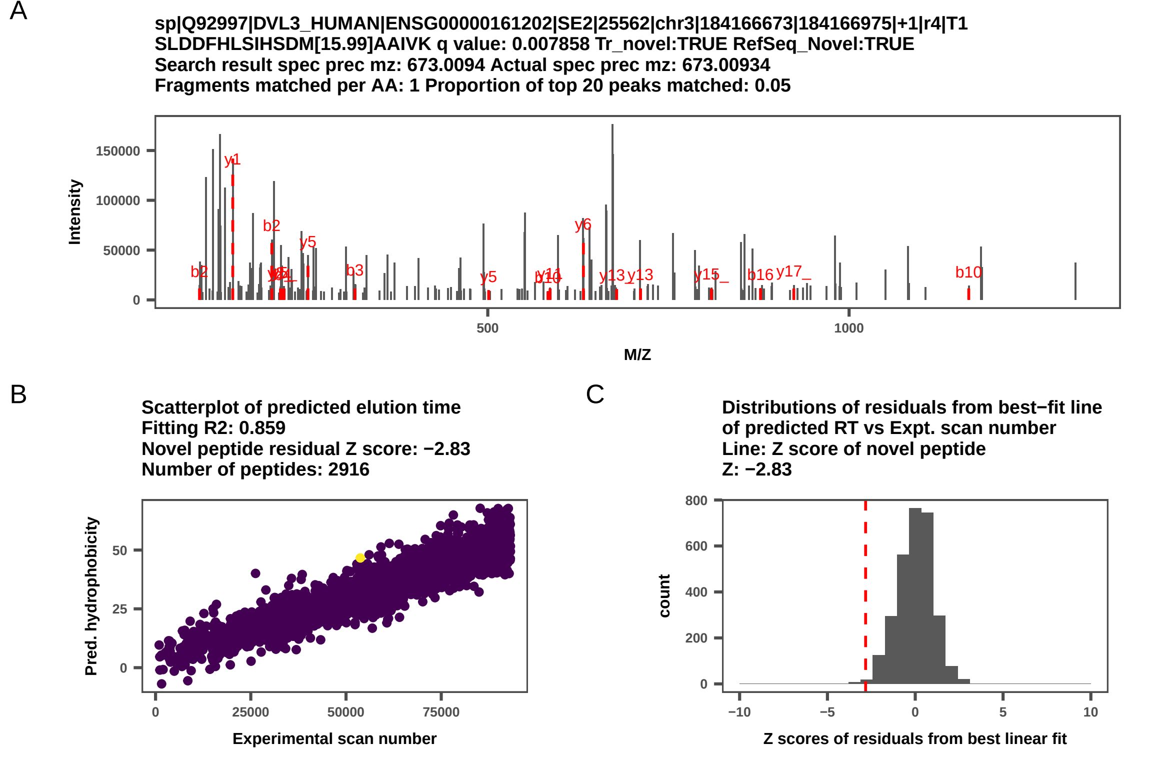
A
sp|Q92997|DVL3_HUMAN|ENSG00000161202|SE2|25562|chr3|184166673|184166975|+1|r4|T1
SLDDFHLSIHSDM[15.99]AAIVK q value: 0.007858 Tr_novel:TRUE RefSeq_Novel:TRUE
Search result spec prec mz: 673.0094 Actual spec prec mz: 673.00934
Fragments matched per AA: 1 Proportion of top 20 peaks matched: 0.05
Intensity
150000
100000
50000
0
M/Z
500
1000
y1
b2
y5
y6
b3
y13
y15_
b16
y17_
b10
B
Scatterplot of predicted elution time
Fitting R2: 0.859
Novel peptide residual Z score: −2.83
Number of peptides: 2916
Pred. hydrophobicity
50
25
0
Experimental scan number
0
25000
50000
75000
C
Distributions of residuals from best−fit line
of predicted RT vs Expt. scan number
Line: Z score of novel peptide
Z: −2.83
count
800
600
400
200
0
−10
−5
0
5
10
Z scores of residuals from best linear fit
